# Carbon monoxide production from five volatile anesthetics in dry sodalime in a patient model: halothane and sevoflurane do produce carbon monoxide; temperature is a poor predictor of carbon monoxide production

**DOI:** 10.1186/1471-2253-5-6

**Published:** 2005-06-02

**Authors:** Christiaan Keijzer, Roberto SGM Perez, Jaap J De Lange

**Affiliations:** 1 Department of anesthesiology, VU University medical center, Amsterdam, The Netherlands

## Abstract

**Background:**

Desflurane and enflurane have been reported to produce substantial amounts of carbon monoxide (CO) in desiccated sodalime. Isoflurane is said to produce less CO and sevoflurane and halothane should produce no CO at all.

The purpose of this study is to measure the maximum amounts of CO production for all modern volatile anesthetics, with completely dry sodalime. We also tried to establish a relationship between CO production and temperature increase inside the sodalime.

**Methods:**

A patient model was simulated using a circle anesthesia system connected to an artificial lung. Completely desiccated sodalime (950 grams) was used in this system. A low flow anesthesia (500 ml/min) was maintained using nitrous oxide with desflurane, enflurane, isoflurane, halothane or sevoflurane. For immediate quantification of CO production a portable gas chromatograph was used. Temperature was measured within the sodalime container.

**Results:**

Peak concentrations of CO were very high with desflurane and enflurane (14262 and 10654 ppm respectively). It was lower with isoflurane (2512 ppm). We also measured small concentrations of CO for sevoflurane and halothane. No significant temperature increases were detected with high CO productions.

**Conclusion:**

All modern volatile anesthetics produce CO in desiccated sodalime. Sodalime temperature increase is a poor predictor of CO production.

## Background

In 1990 first reports were published about carbon monoxide (CO) production in anesthetic circuits [1-3] followed by a few studies that concluded that there was no risk of CO intoxication in common anesthetic practice [4-6]. The potential risk of CO production, however, was clearly established in a laboratory study by Fang et al. [7]. This study was the first to prove that desflurane produced higher amounts of CO compared to enflurane and isoflurane respectively, when in contact with dry sodalime and Baralyme^®^. Furthermore, they found that Baralyme^® ^produced higher amounts of CO compared to sodalime with all three volatile anesthetics. Frink et al. [8] and Bonome et al. [9] demonstrated in animal studies that desflurane produced high amounts of CO in dry carbon dioxide absorbents, with higher amounts in Baralyme^® ^than sodalime.

In an in vitro study, Wissing et al[10] found high concentrations of CO production for enflurane and isoflurane as well, and to a lesser extent for sevoflurane and halothane. Wissing et al. further found temperature increase in all analyzed volatile anesthetics, which has been linked to higher production of CO [7]. However, as this study was performed using only a gas flow over a carbon dioxide absorber canister, these results cannot easily be extrapolated to a clinical situation. Furthermore CO measurements were performed with infrared absorption and electrochemical detection which are not as accurate as gas chromatography [11].

Therefore, the purpose of this study is to measure in a simulated patient model, the maximum amounts of CO production for all modern volatile anesthetics, with completely dry sodalime using a gas chromatograph. Also, temperature of the system was measured to establish the relationship between CO production and temperature increase.

## Methods

### Patient model

Two sample lines were connected to the Y-piece of the circle system: one to a small lumen gas chromatography sample line connected to the gas chromatograph, and one connected to the infrared anesthetic vapor analyzer (SAM, Marquette) sampling at 200 ml/min.

The volatile anesthetics and the sodalime (Drägersorb^® ^800 plus, composition: 0.003% KOH, 2% NaOH, 82% Ca(OH)_2 _and 16% H_2_O) were obtained from our own stock. The sodalime was dried completely by using an oxygen flow of 15 l/min in sealed glass containers until no more weight reduction could be measured. A 16% weight reduction was established confirming the producer's specifications.

### Experiments

For each anesthetic vapor, an experiment was performed in which 950 grams of dry sodalime was used. The ventilator was set in IPPV mode with a tidal volume of 600 ml, a frequency of 14/min and 5 cm H_2_O PEEP. After an equilibration with 40 % oxygen and 60% nitrous oxide was established at a fresh gas flow (FGF) of 5 l/min, anesthetic vapor was introduced by a standard vaporizer. The dial was set until the vapor analyzer showed the target concentration of anesthetic vapor, after which the FGF was reduced to 500 ml/min. For the different anesthetic vapors equilibrium was maintained of 0.45 vol% halothane, 0.6% enflurane, 0.6% isoflurane, 0.8% sevoflurane and 3.0% of desflurane during an experiment.

### Carbon monoxide measurements

A portable gas chromatograph (Varian Chrompack CP 2003P) with a TCD detector and a Mollsieve 5A column was used for CO quantification with a lower limit of 1 ppm. This gas chromatograph (GC) is capable of automatic sampling and was programmed to sample approximately every five minutes during an experiment (a total of 36 samples). The GC was calibrated with two calibration mixtures of 210 and 981 parts per million (ppm) CO in nitrogen (Hoekloos specialty gasses, Dieren). The GC was connected to a desktop PC for control of the GC and data recording, analysis and storage.

### Temperature measurements

The sodalime container of the circle system was equipped with temperature probes in the upper and lower layer of the container, temperature data were continuously recorded (samplefrequency 30 Hz) during each experiment.

### Analysis of data

The total amount of CO production and absorbent temperature were measured for each of the five volatile anesthetics. All experiments were performed in duplicate (ten experiments in total) in order to verify the reproducibility of the CO measurements. To verify that no CO was produced in normal circumstances, i.e. with fresh sodalime, these measurements were repeated with fresh sodalime.

Analyses were performed with SPSS 11.0. The Mann-Whitney-U was used to assess the reproducibility CO measurements, expressed as lack of significant differences between consecutive measurements, the Kruskal-Wallis and Mann-Whitney-U tests for comparison of CO productions between volatile anesthetics, and temperature change. Data were presented as peak, median and IQR (interquartile range) CO concentrations. For all analyses the significance level was set at 5%.

## Results

### Carbon monoxide measurements

When the first measurements started different amounts of CO were measured when the sodalime was not sufficiently dried. Therefore each experiment was performed twice and no significant difference was found between consecutive measurements. P-values were for desflurane, enflurane, isoflurane, halothane and sevoflurane respectively: 0.906, 0.481 . 1.00, 0.839, 0.725.

The control experiments with fresh sodalime showed no CO production.

**Figure 1:**
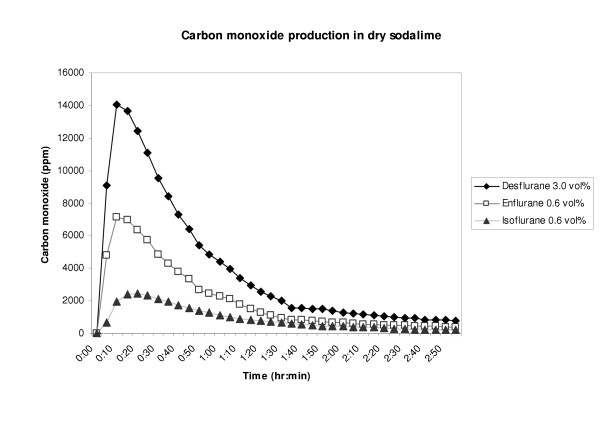
Carbon monoxide production of desflurane, enflurane and isoflurane in desiccated sodalime. Legend: Carbon monoxide was measured in parts per million (ppm).

**Figure 2:**
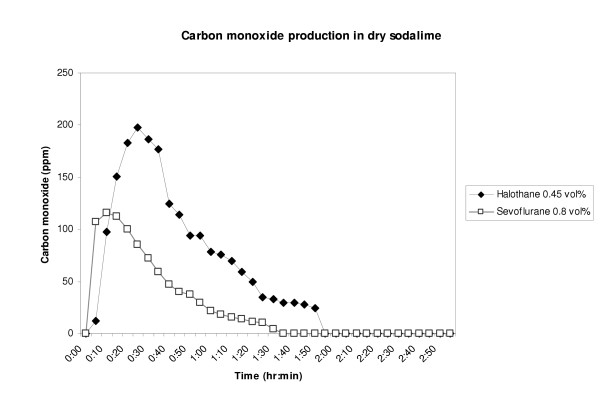
Carbon monoxide production of halothane and sevoflurane in desiccated sodalime. Legend : Carbon monoxide was measured in parts per million (ppm),

Mean CO concentrations measured by the GC were calculated for each anesthetic vapor. Figure 1 shows the CO concentration for desflurane, isoflurane and enflurane. Because of distinctly lower CO productions for halothane and sevoflurane, both measurements were depicted in a separate figure (figure 2). A fast increase of CO concentration is seen with shortly after a slow exponential like decrease in concentration.

Complete peak, median and interquartile range (IQR) CO concentrations of all experiments are shown in table 1. Highest CO concentrations in parts per million (ppm) were measured with peak concentrations of 14262 ± 694 for desflurane, followed by 10654 ± 510 for enflurane, 2512 ± 126 for isoflurane and 210 ± 11 for halothane and 121 ± 7 for sevoflurane. Significant differences were found between CO production of the five volatile anesthetics (Kruskall Wallis: p < 0.001). Except for the comparison between desflurane – enflurane (Mann-Whitney-U: p = 0.303) and halothane – sevoflurane (Mann-Whitney-U: p = 0.079) all paired comparisons were significantly different (Mann-Whitney-U: all p < 0.001)

**Table 1:** Carbon monoxide concentrations. Legend: Peak and median interquartile range carbon monoxide concentration [CO] in parts per million for each experiment: ex.1 = experiment 1, ex.2= experiment 2, both with desflurane 3.0 vol%, enflurane 0.6 vol%, isoflurane 0.6 vol%, halothane 0.45 vol% and sevoflurane 0.8 vol% in completely dry sodalime.

Anesthetic vapor	Peak [CO] ex.1	Peak [CO] ex.2	Median [CO] ex.1	Median [CO] ex.2
Desflurane	13889	14262	1809 (1092–5947)	1816 (1050–6378)
Enflurane	10187	10654	1485 (793–4490)	2044 (892–4394)
Isoflurane	2512	2382	588 (329–1430)	664 (329–1311)
Halothane	185	210	28 (0–92)	31 (0–94)
Sevoflurane	113	121	0 (0–36)	5 (0–43)

### Temperature measurements

The temperature measurements at the bottom of the sodalime container showed a mean temperature rise from 23.5 to 28.3°C in the experiments with fresh sodalime. In the experiments with dry sodalime (except for sevoflurane) a rise in mean temperature from 24.0 to 32.9°C was measured.

In the experiments with dry sodalime and sevoflurane a high increase in temperature from 26.0 to 67.7°C was measured during the first twenty minutes. In those twenty minutes the sevoflurane dial had to be set at maximum because otherwise 0.85 vol% sevoflurane could not be maintained in the circle system.

In all experiments a small difference in temperature was seen between the upper and lower layer of the sodalime container with a slightly higher temperature of 0.8 – 1.0°C at the bottom of the container.

## Discussion

### Carbon monoxide production

For this study we developed a method in which a gas chromatograph sampled automatically every five minutes during each experiment, therefore providing the most accurate and reliable CO measurement. To our knowledge this is the first time this kind of setup was used.

In this study the findings of Fang et al[7] concerning the fact that desflurane produces more CO than enflurane and isoflurane respectively, were confirmed. However, instead of using small vials of 30 ml, we used a patient model, therefore measuring the maximum amounts of CO in completely dry sodalime at a concentration equivalent of approximately 1 MAC of volatile anesthetic using a oxygen/nitrous oxide mixture. Regarding the toxicity of CO, the Henderson and Haggard's Index of Toxic effect[12] indicates that one hour of exposure of more than 1500 ppm of CO is dangerous to life. However one should also take into consideration that the CO is not continuously produced in this model in contrast with this index and that CO absorption by a patient is not included in this model. Therefore we can only conclude from our findings that in these extreme conditions very high CO concentrations can be reached for desflurane and enflurane and that isoflurane can produce significant concentrations of CO as well. One should take into consideration that the use of Baralyme^® ^will produce higher levels of CO[7,13], and that carbon dioxide absorption, fresh gas flow and minute volume have small effects on CO production as shown by Woehlck et al. [13]. Because of the relative small effect of carbon dioxide absorption on CO production we didn't add carbon dioxide to our model.

As for the clinical relevancy, one could say that this model uses completely dry sodalime which is not seen very frequently in common anesthetic practice. However, there are reports of severe CO intoxications [2,3] recently published by Berry et al. [14] with desflurane as anesthetic agent. The highest risk develops when fresh gas flow is maintained in a anesthesia system for a few days. After 41 hours with a 7 l/min fresh gas flow, the soda lime will become critically dry as published by Soro et al. [15]. As there is always a potential risk, one should consider a safety protocol to maintain a proper humidity level inside the carbon dioxide absorbent as proposed by Woehlck et.al [16], especially when using anesthetic agents like desflurane and enflurane. One could also consider the use of more accurate electrochemical CO monitors [17,18] which can detect CO by continuous measuring in the anesthetic circuit. Another possibility is the use of different carbon dioxide absorbents, particularly absorbents with less Ba(OH)_2_, KOH and NaOH [19,20] that produce relatively safe amounts of CO or have no CO production at all[21,22].

During the desflurane experiments the infrared anesthetic vapor analyzer reported a concentration of enflurane up to 1.0 vol%, which correlated significantly with the measured CO concentration (Spearman's r: 0.805; p < 0.001). This reported enflurane concentration is probably attributable to the production of trifluoromethane that is simultaneously produced with CO[23] and is known to be detected as enflurane by this vapor analyzer[24]. The enflurane detection disappears below a CO concentration of 3400 ppm, which explains why in the isoflurane experiments no 'enflurane' was detected. In case of a 'mixed gas' warning or a unexpected 'enflurane' detection during anesthesia using desflurane, one should consider the possibility of a (high) CO production.

Contrary to reports in literature[7], we found significant amounts of CO with halothane and sevoflurane. Also CO production by both substances is not explained by the mechanism postulated by Baxter et al[23]. Previously, CO production was reported by Strauss et al. [25] for halothane and Wissing et al[10] for both sevoflurane and halothane. They reported higher concentrations of CO than found in our study, but at higher concentrations of these two volatile anesthetics and with use of a KOH containing absorber. Our reported amounts of CO are not dangerous for several hours in healthy individuals, but could be clinically relevant for anemic patients or small children[26,27].

### Temperature measurements

No clinically relevant temperature increase was measured during the experiments with dry sodalime and desflurane, enflurane, isoflurane and halothane. This is not concurrent with findings of other authors [10,28]. Our explanation for these differences is the use of higher concentrations of vapor and a higher fresh gas flow used in the experiments of these studies which would give a more exothermic reaction than in our study. We did however measure a forty degrees Celsius temperature increase in the experiments with sevoflurane and dry sodalime. Simultaneously, we noticed a high degree of sevoflurane degradation because of the discrepancy between dial setting of the vaporizer and the measured sevoflurane concentrations in the circle system. This confirms the report of instability of sevoflurane in desiccated sodalime by Funk et.al[29]. We concluded that temperature measurement in the sodalime container is a very poor predictor of CO production because of the high CO production with desflurane with a small increase of temperature and the other way round for sevoflurane. However a study from Holak et.al. [27] demonstrated that clinically relevant CO concentrations with the use of Baralyme^® ^do not occur until the absorbent temperature exceeds 80°C. Because of the use of a combination of sevoflurane and nitrous oxide in this study we cannot rule out that higher concentrations of sevoflurane without nitrous oxide would increase the absorbent temperature above a certain threshold where sodalime could also be capable of production of high concentrations of CO or even result in fire or explosions as recently reported with the use of dessicated Baralyme^® ^and sevoflurane [30-32]. Further studies using sevoflurane and other absorbents with temperature measurement inside the absorbents [33] should be performed to determine if these reactions can also occur with other absorbents than Baralyme^®^.

## Conclusion

In this patient model we demonstrated the possible production of very high amounts of CO in dry sodalime with desflurane and enflurane. CO production from isoflurane is less but still significant. Also sevoflurane and halothane can produce small amounts of CO. A report from the vapor analyzer that a mixed gas or a certain amount of enflurane is present when using desflurane suggests that more then 3400 ppm CO is already present in the anesthesia circle system.

When using desflurane one should consider implementing a safety protocol to prevent the sodalime from completely drying out. Another option is the choice for a 'safer' carbon dioxide absorber. Measurement of the soda lime temperature is a poor predictor for CO production in sodalime when using anesthetic vapor in combination with nitrous oxide.

## Competing interests

The author(s) declare that they have no competing interests.

## Authors' contributions

CK participated in the design of the study, performed all experiments, participated in the statistical analysis and drafted the manuscript. RP participated in the statistical analysis and helped to draft the manuscript. JL participated in the design of the study and helped to draft the manuscript. All authors read and approved the final manuscript.

## Pre-publication history

The pre-publication history for this paper can be accessed here:


